# Perspective: advancing public health education by embedding AI literacy

**DOI:** 10.3389/fdgth.2025.1584883

**Published:** 2025-07-16

**Authors:** Jose A. Acosta

**Affiliations:** School of Medicine, Ponce Health Sciences University, Ponce, Puerto Rico

**Keywords:** artificial intelligence, public health education, curriculum development, digital health literacy, ethics

## Abstract

Artificial intelligence (AI) fundamentally reshaping public health practice, yet formal training in AI literacy remains scarce in most public health educational programs. The rapid emergence of large language models and other AI-driven technologies such as computer vision, predictive analytics, and natural language processing tools—used in applications ranging from epidemiological modeling and policy analysis to real-time health communication—highlights the urgent need to bridge a persistent knowledge gap in structured, competency-based AI training for public health students and professionals. This *Perspective* article introduces the growing role of AI in public health, examines challenges in diverse global settings, outlines current gaps in AI literacy training, and proposes a framework for integrating AI competencies into undergraduate, graduate, and continuing public health curricula. In doing so, it emphasizes the importance of equipping tomorrow's public health workforce with the ethical, technical, and critical-thinking skills needed to harness AI's potential to improve health outcomes and support public health practice across diverse and underserved communities.

## 1 Introduction and rational

Artificial Intelligence (AI) is transforming nearly every facet of public health, from disease surveillance to policy development (1). Large language models (LLMs) such as ChatGPT, Claude, DeepSeek, Llama, Co-Pilot and Gemini are now employed in epidemiological modeling, crisis response, and health communication, offering powerful new tools for data-driven decision making (2). While these tools show considerable promise, most LLMs remain experimental in public health settings, and their integration into real-world practice is still limited.

However, AI literacy—defined as the ability to understand, critically evaluate, and responsibly apply AI—has not been systematically integrated into public health education (3). Without structured instruction, students risk disseminating misinformation, over-relying on AI-generated insights, and overlooking critical ethical implications. Recognizing the significance of AI in advancing global health objectives, the World Health Organization (WHO) has highlighted the need for AI literacy as a core competency for the next generation of healthcare professionals (4). This *Perspective* article argues that embedding AI-focused curricula into undergraduate, graduate, and continuing education public health programs is both timely and critical, ensuring that future practitioners can navigate the AI-driven public health landscape responsibly.

## 2 The expanding role of AI in public health

### 2.1 Advances in epidemiology and policy

AI-driven algorithms, particularly through advanced nowcasting techniques and foundation models, have markedly improved real-time disease forecasting, precise risk stratification, and rapid outbreak detection (5). By leveraging graph neural networks and multimodal machine learning, these tools can accurately identify transmission clusters, integrate complex risk factor data, and assess the effectiveness of public health interventions.

In addition, LLMs enable the rapid development of data-driven policy briefs, tailored health communications, and early detection of misinformation, advancing the effectiveness and equity of large-scale public health initiatives. For instance, in Chaco, Argentina, a behaviorally informed AI-powered chatbot increased COVID-19 vaccine uptake in underserved communities by more than three times compared to control groups (6). Despite such a promising example, successfully deploying AI-driven solutions in resource-limited settings requires addressing significant implementation challenges, including limited digital infrastructure, a shortage of trained personnel, and fragmented data governance systems.

### 2.2 Applications in resource-limited settings

Many low- and middle-income countries face structural barriers to AI integration, including limited internet access, trained personnel shortages, and financial constraints (7). Despite these challenges, context-appropriate AI solutions are emerging worldwide. India's e-Sanjeevani 2.0 platform demonstrates how telemedicine powered by basic AI triaging can serve millions without requiring sophisticated infrastructure (8). Similarly, UNICEF's Magic Box initiative employs AI and mobile data to track population displacement during emergencies in sub-Saharan Africa, supporting targeted public health responses (9). These examples illustrate how thoughtfully designed AI applications can promote health equity and deliver meaningful impact, even in resource-constrained settings. Success depends on tailoring solutions to local needs and infrastructural realities, rather than transplanting high-resource models.

### 2.3 AI in imaging and diagnostics

AI has demonstrated significant progress in image recognition and classification, as shown in a recent study evaluating deep learning models for monkeypox detection. Among the models tested, MobileNetV2 achieved the highest accuracy at 98.16%, outperforming other pretrained architectures such as VGG19, VGG16, ResNet50, and EfficientNetB3 (10). In clinical laboratory settings, AI has been linked to enhanced diagnostic accuracy and workflow efficiency, enabling faster pathogen detection and more precise, data-driven treatment decisions (11). These findings highlight the potential of AI-powered diagnostics for early detection and timely treatment, which are critical factors in reducing infectious disease morbidity and mortality. Taken together, these applications underscore AI's growing utility across public health functions, setting the stage for a deeper examination of how academic institutions can prepare students for this evolving landscape.

## 3 Gaps in AI literacy training

Despite these advances, most public health programs do not offer structured AI coursework, leaving students to acquire these skills through informal or *ad hoc* methods (12, 13). Key learning deficits include the lack of exposure to basic AI principles, such as machine learning and neural networks, and the scarcity of hands-on experience with epidemiological modeling, policy simulation, or data analysis using AI-driven tools. Additionally, students seldom receive formal instruction on AI bias, privacy, and responsible data governance, which inhibits their ability to mitigate risks (14).

Faculty hesitancy remains a significant barrier to AI adoption in higher education. Surveys cited in recent literature report that while a majority of faculty acknowledge AI's educational potential, a substantial proportion still lack confidence or formal knowledge about AI technologies, highlighting a critical gap in faculty preparedness for AI integration (15). Additionally, a WGU Labs study found that only 42% of faculty believe AI tools will positively impact their roles, and 49% did not use them in the classroom (16).

Promising examples of structured implementation do exist. The University of Helsinki's “Elements of AI” initiative provides a free, open-access AI literacy course for students and professionals, including those in public health. With over 1,000,000 learners worldwide, this program demonstrates how AI education can scale in non-technical fields without extensive resource investment (17).

## 4 A framework for AI literacy integration

To bridge the growing gap between AI advancements and current public health education, schools are encouraged to embed AI training into their core curricula, a recommendation supported by the World Health Organization and echoed in recent frameworks on digital health education and biomedical science training (4, 15). The first pillar focuses on technical foundations, introducing basic principles of machine learning, data science, and natural language processing to equip students with an understanding of AI functionality and applications in public health contexts. The second pillar addresses ethical and regulatory literacy, developing critical evaluation skills through case studies highlighting issues such as algorithmic bias, data misuse, and privacy concerns, helping students navigate complex legal and social implications.

The third pillar emphasizes experiential learning, engaging students in hands-on projects like disease forecasting or policy simulation, ideally through interdisciplinary collaboration with computer science departments. The fourth pillar examines governance and policy, exploring how AI shapes health policy development, influences public trust, and demands social accountability through real-world implementation examples. The final pillar centers on equity and access, addressing financial and infrastructural realities, particularly in low-resource settings, and promoting open-source tools and sustainable implementation models.

**Figure 1:**
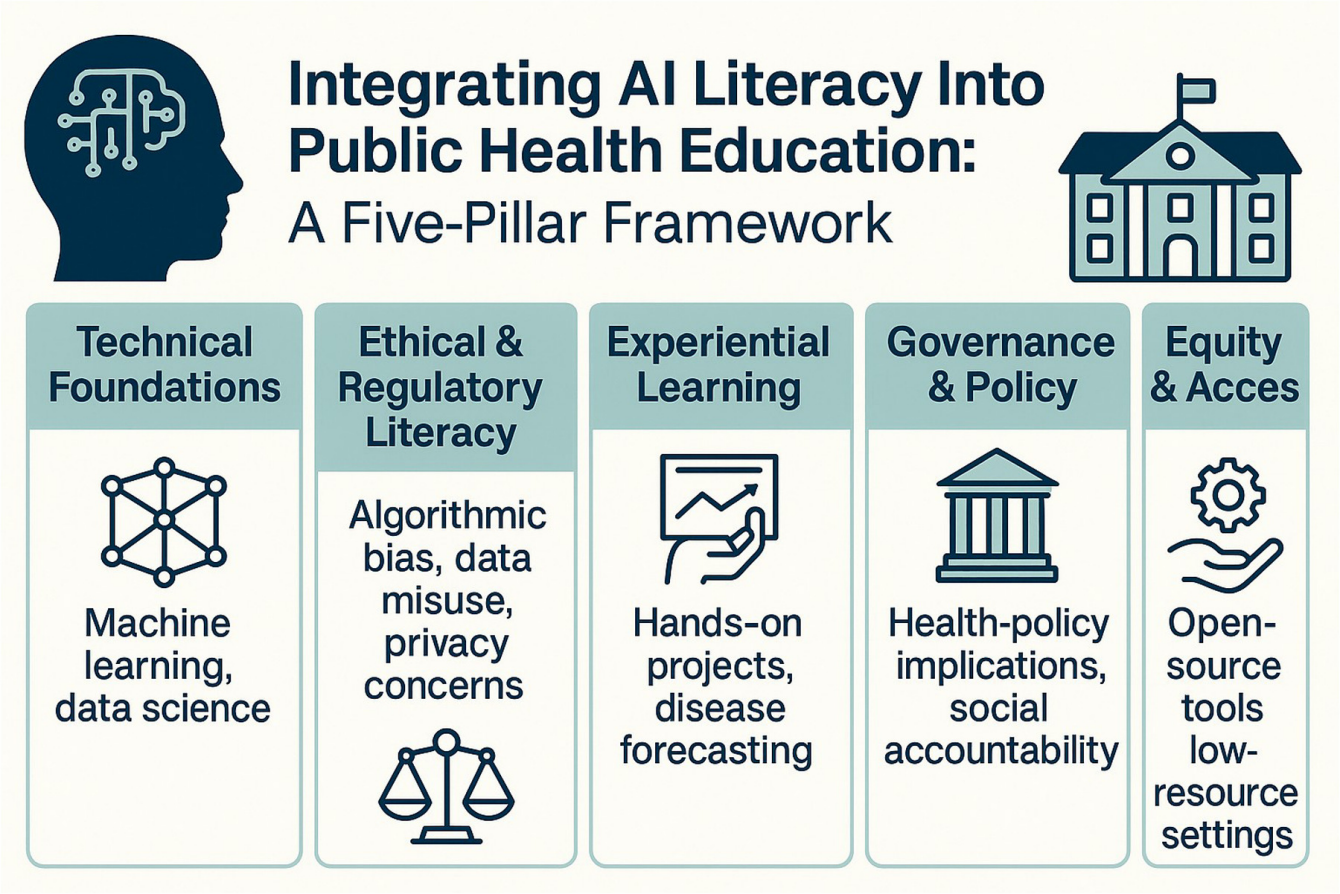
Conceptual model showing five pillars—technical basics, ethics/regulation, hands-on learning, policy governance, and equitable access—that embed AI literacy in public-health education.

The five-pillar framework presented in this *Perspective* article is a synthesized model developed by the author to organize core domains of AI literacy in public health education (see Figure 1). It draws conceptually from recurring themes in expert guidance, implementation literature, and educational policy—including technical foundations, ethics and regulation, experiential learning, governance and policy, and equity and access—but has not been formally published in this unified form (3, 4, 7, 13–15, 17, 18).

Realizing this framework, however, depends not only on curriculum design but also on the readiness and support of those responsible for delivering it.

## 5 Overcoming barriers: faculty development and institutional support

Faculty readiness, institutional policies, and accessible resources collectively determine whether AI literacy initiatives can succeed. Many educators remain uncertain about effectively employing AI in teaching or distinguishing between responsible uses and potential pitfalls (15, 19).

To support faculty in adopting AI, universities can offer short courses and workshops that build foundational skills through hands-on learning. Collaborations with industry partners and opportunities for research or sabbaticals can deepen faculty expertise. Institutions should also establish clear policies on academic integrity and responsible AI use, which can alleviate concerns about misuse. Providing open-access tools and shared licenses helps reduce cost barriers, making integration more feasible. When faculty feel equipped and supported, they are more likely to innovate and guide students effectively, fostering a collaborative, forward-looking academic environment.

## 6 Ethical and equity considerations

The integration of AI into public health demands careful attention to ethical and equity considerations, with rigorous benchmarking of AI models serving as a cornerstone for ensuring transparent, trustworthy, and responsible applications in healthcare (20). Data privacy and security concerns must be addressed early in the educational process, particularly as many AI models depend on large health databases containing sensitive patient information. Beyond privacy, algorithmic bias poses a significant risk, as AI models can inadvertently amplify existing social inequalities if trained on unrepresentative datasets (18). Striking the right balance between AI-driven innovation and the safeguarding of patient safety and data integrity necessitates proactive oversight and deliberate governance (21).

Public health curricula must include training in bias detection and mitigation strategies, nuanced discussion of model validation and performance evaluation across diverse populations. By recognizing that AI adoption can exacerbate social inequities if not carefully managed, the next generation of public health professionals will be better equipped to advocate for ethical, inclusive solutions.

## 7 Limitations and future directions

This *Perspective* article has several limitations that warrant acknowledgment. First, as a conceptual piece, it lacks comprehensive empirical data on AI literacy implementation in public health curricula. The framework does not currently include methods for evaluating learning outcomes or assessing competencies related to AI literacy. Pilot testing and validation in diverse educational settings will be important to determine its feasibility, effectiveness, and adaptability.

Second, the rapid evolution of AI technologies and their applications in healthcare suggests that any proposed framework must remain flexible. Ongoing adaptation will be essential to respond to emerging innovations, evolving competencies, and dynamic regulatory landscapes. What constitutes essential knowledge today may shift as capabilities advance and new ethical and practical challenges arise.

Third, while this article attempts to incorporate global perspectives, the illustrative examples are limited and may not fully capture the breadth of implementation contexts across high-, middle-, and low-resource settings. Future work should aim to document and evaluate AI-related training strategies in a variety of educational, cultural, and infrastructural environments.

Finally, the current framework is focused primarily on public health education programs. Many AI competencies—such as ethical reasoning, data interpretation, and interdisciplinary collaboration—are equally relevant across the broader landscape of interprofessional education, including medicine, nursing, pharmacy, and health informatics. Expanding the framework's relevance across disciplines may enhance its utility and promote more integrated workforce readiness.

## 8 Conclusion

AI-driven solutions are rapidly transforming the fundamental operations of public health, encompassing the diagnosis of infectious diseases and the management of extensive interventions. Yet formal AI training remains absent in most programs, creating a critical gap that leaves students and professionals unprepared for data-driven decision-making. By identifying knowledge deficits, barriers to adoption, and strategies for overcoming them, this article offers a perspective for integrating AI literacy into public health curricula globally.

Given the WHO's emphasis on AI literacy for global health professionals, the time for educational reform is now (4). Through coordinated efforts to build comprehensive AI literacy—grounded in the five pillars of technical foundations, ethical and regulatory literacy, experiential learning, governance and policy, and equity and access—public health schools can equip the next generation of leaders with the competencies needed to safeguard communities and enhance health outcomes in the digital age.

## Data Availability

The original contributions presented in the study are included in the article, further inquiries can be directed to the corresponding author.
